# Cardiovascular outcomes of type 2 diabetic patients treated with DPP‑4 inhibitors versus sulphonylureas as add-on to metformin in clinical practice

**DOI:** 10.1038/s41598-021-02670-9

**Published:** 2021-12-13

**Authors:** Juan Carlos Bazo-Alvarez, Kingshuk Pal, Tra My Pham, Irwin Nazareth, Irene Petersen, Manuj Sharma

**Affiliations:** 1 Research Department of Primary Care and Population Health, University College London (UCL), Rowland Hill Street, London, NW3 2PF UK; 2 Escuela de Medicina, Universidad Cesar Vallejo, Trujillo, Peru; 3 MRC Clinical Trials Unit at UCL, London, UK

**Keywords:** Cardiology, Endocrinology

## Abstract

DPP-4 inhibitors (DPP-4i) and sulphonylureas remain the most widely prescribed add-on treatments after metformin. However, there is limited evidence from clinical practice comparing major adverse cardiovascular events (MACE) in patients prescribed these treatments, particularly among those without prior history of MACE and from vulnerable population groups. Using electronic health records from UK primary care, we undertook a retrospective cohort study with people diagnosed type-2 diabetes mellitus, comparing incidence of MACE (myocardial infarction, stroke, major cardiovascular surgery, unstable angina) and all-cause mortality among those prescribed DPP-4i versus sulphonylureas as add-on to metformin. We stratified analysis by history of MACE, age, social deprivation and comorbidities and adjusted for HbA1c, weight, smoking-status, comorbidities and medications. We identified 17,570 patients prescribed sulphonylureas and 6,267 prescribed DPP-4i between 2008–2017. Of these, 16.3% had pre-existing MACE. Primary incidence of MACE was similar in patients prescribed DPP-4i and sulphonylureas (10.3 vs 8.5 events per 1000 person-years; adjusted Hazard Ratio (adjHR): 0.94; 95%CI 0.80–1.14). For those with pre-existing MACE, rates for recurrence were higher overall, but similar between the two groups (21.8 vs 17.2 events per 1000 person-years; adjHR: 0.93; 95%CI 0.69–1.24). For those aged over 75 and with BMI less than 25 kg/m^2^ there was a protective effect for DPP-I, warranting further investigation. Patients initiating a DPP-4i had similar risk of cardiovascular outcomes to those initiating a sulphonylurea. This indicates the choice should be based on safety and cost, not cardiovascular prognosis, when deciding between a DPP-4i or sulphonylurea as add-on to metformin.

Despite emergence of several novel therapies, dipeptidyl-peptidase-4 Inhibitors (DPP-4i) and sulphonylureas still remain the most commonly used add-on therapy to metformin^1,2^. Studies have consistently demonstrated similar glycaemic reductions with both treatments when added to metformin, and a higher hypoglycaemic risk with sulphonylureas^3,4^. However evidence around long-term complications, in particular cardiovascular safety, has only begun to emerge more recently^5,6^. Cardiovascular events account for 70% of deaths in patients with type 2 diabetes mellitus (T2DM)^4,7^, and in 2008 the FDA introduced legislation that made it mandatory to undertake cardiovascular safety studies with novel anti-diabetics^5,8,9^. Results from several of these trials are now available for commonly used DPP-4i^9^.

The results from cardiovascular safety trials with DPP-4i suggest they do not increase risk of major adverse cardiovascular events (MACE), compared to placebo, or compared to sulphonylureas in the CAROLINA trial^5,10^. However despite randomised controlled trials being the gold standard in evaluating efficacy and safety, they are sometimes prone to selection bias by recruiting participants not reflective of the clinical practice, which compromises their external validity^11^. Real world studies have been undertaken, but thus far have often been limited by sample size, inadequate comparators, short follow-up or been unable to adjust for important confounding compromising accuracy of estimates^12–14^. Several studies have also been unable to explore outcomes among vulnerable groups such as older adults, those with established cardiovascular disease and those from poor socioeconomic backgrounds. Therefore, the risk of developing long-term cardiovascular complications of T2DM with DPP-4i, still requires further evidence from clinical practice.

Equally, the impact of use of sulphonylureas on cardiovascular events remains uncertain, with studies demonstrating both benefit as well as increased risk^10,15^. A recent meta-analyses of randomised controlled trials and observational studies involving sulphonylureas suggested that overall the risk of cardiovascular events and mortality is higher in patients with T2DM treated with sulphonylureas versus other types of glucose-lowering agents (including DPP-4i), however studies included were highly heterogenous^16^.

In this study, we used primary care data to compare incidence of MACE with DPP-4i against sulphonylureas in people diagnosed T2DM. We examine the outcome for patients both with and without a history of MACE, and stratified across several groups including by age, socioeconomic status and key clinical characteristics.

## Methods

### Data source and cohort identification

Electronic health records from the IQVIA Medical Research Data (IMRD) UK primary care database were used. IMRD contains records from 711 general practices in the UK collected during routine patient consultations. This includes information on demographic, diagnostic, prescribing and clinical indicators from around 12 million patients. IMRD uses the hierarchical Read Code system, which is a standardised coding system for recording clinical data. IMRD has been shown to be representative of the UK population^17,18^. We focused our analysis on practices in IMRD that met key quality standards related to acceptable computer usage and reported mortality rates consistently with national statistics.

Within the IMRD database, we identified individuals with T2DM, prescribed a DPP-4i or sulphonylurea as add-on to metformin between 2008–2017, using an algorithm previously validated^19^. The date on which the first prescription for either DPP-4i or sulphonylurea was added-on was the index date. We required that they were aged 18–99 years at the day of first prescription, registered with a general practice permanently, prescribed metformin as add-on (not replacement), and had a minimum of 12 months data recorded at baseline. We excluded T2DM patients with a history of any other anti-diabetic drug (other than metformin) prior to second-line treatment initiation, and with missing data on key covariates namely, HbA1c, systolic blood pressure and weight at add-on initiation.

### Outcome definitions

Our primary outcome was major adverse cardiovascular events (MACE) which included a composite of myocardial infarction, stroke, diagnosis of unstable angina and major cardiovascular surgery, with a view to mirroring the completed trials as closely as possible. As linkage to hospital records was not possible, hospitalisation for unstable angina was not included as part of our MACE definition, hence this amendment was used. Major cardiovascular surgery included surgery that is routinely undertaken for myocardial infarction, stroke and major cardiovascular disease was treated as a separate category, as these individuals did not have a diagnostic Read code to confirm cardiovascular diagnosis. Equally, due to well-documented difficulty in ascertaining causes of death accurately in IMRD^20^, it was not possible to ascertain cardiovascular death rates accurately to include in the main outcome definition. Instead we examined all-cause mortality which is robustly captured^20^, as a separate, secondary outcome. Additionally, we also ran sensitivity analysis, to determine if inclusion of all-cause mortality as part of the MACE definition itself, impacted on results.

### Statistical analysis

General baseline characteristics were obtained from measurements made in the 12 months prior to add-on treatment initiation. These characteristics included potential confounders that were considered in the main analyses (i.e., adjusted estimates): age, sex, socioeconomic status (Socioeconomic status, with Townsend score), baseline HbA1c, history of hypoglycaemia, systolic blood pressure, weight, history of chronic kidney disease, severe mental illness (SMI), smoking, and prescribing of statins, anti-hypertensives, antianginals, anti-heart-failure, lipid lowering, anti-platelets, anti-coagulates, anti-depressants, anti-psychotics, anti-obesity, steroids, thyroxine, anti-thyroid and anti-arrythmic medication. MACE was defined identically when considered as part of baseline characteristics (for those with a previous history of MACE) and as part of our outcome. SMI included bipolar disorder, schizophrenia and non-organic psychotic disorders all established as major risk factors for cardiovascular events^21^. All disease and drug covariates were marked as present (binary variables) if a record indicative of the disease in medical or additional healthcare records was registered any time prior to the add-on treatment initiation.

For our unadjusted analysis, we followed both DPP-4i and sulphonylurea cohorts from first prescription until first incidence of MACE (first subsequent occurrence (secondary incidence) in those with history of MACE), censoring on the date individuals left the general practice, died or the observation period finished. We fitted Cox proportional-hazard regression models and used their survival predictions for visualising the trajectory of both cohorts, overall and stratified by history of MACE (yes/no). Unadjusted hazard ratios (HR) allowed the risk comparison between DPP-4i and sulphonylurea cohorts. Kaplan-Meir curves with risk tables and Log-rank tests for the same follow up are also provided. For our adjusted analysis, we compared risk of MACE between DPP-4i and sulphonylurea cohorts by using HR estimated from Cox proportional-hazard regression models as well, adjusted for covariates (described above). We performed a sensitivity analysis combining all-cause mortality with MACE (composite outcome) to evaluate if inclusion of mortality in the primary MACE outcome impacted on findings.

We fitted similar survival models for each of the four components of MACE and all-cause mortality, overall and stratified by history of MACE (yes/no). Additional subgroup analysis with similar Cox models were performed for comparing risk of MACE between DPP-4i and sulphonylurea cohorts, by stratifying the overall sample by age (< 75 vs ≥ 75 years), socio-economic status (SES, 2 least vs 3 most deprived quintiles), body mass index (BMI, < 25 vs ≥ 25 kg/m2), HbA1c (7.5% (58 mmol/mol) and 9.0% (75 mmol/mol) as cut-off points), SMI (yes/no) and chronic kidney disease (CKD yes/no). For Cox models, we evaluated the proportional-hazard assumption graphically and with a global test. All estimates were given with 95% confidence intervals (C.I.) and the statistical analyses were performed using Stata/MP version 16 for Windows (StataCorp. 2019. Stata Statistical Software: Release 16. College Station, TX: StataCorp LLC, https://www.stata.com/statamp/).

### Ethical approval

THIN data, also known as IQVIA Medical Research Data, have a REC Reference 18/LO/0441 as visible in the NHS Health Research Authority website (here). Scientific approval to undertake this study was received from the South East Medical Research Scientific Review Committee at IQVIA (SRC Reference Number: 18THIN003). The IQVIA SRC did not request extra participants consent for this study, and IQVIA counts with all permissions requested by the NHS Health Research Authority (including waiver of consent). All research methods were carried out in accordance with the NHS Health Research Authority guidelines and regulations.

## Results

### Patient disposition and characteristics

In total, we identified 23,387 patients prescribed either a DPP-4 inhibitor (6,267) or sulphonylurea (17,570) as add-on therapy to metformin between 2008–2017, as illustrated in Appendix A.

Within the cohort of 23,837 individuals, 17,570 were prescribed a sulphonylurea and 6,267 and DPP-4i. The mean age was similar in both groups, 60 years among those prescribed sulphonylureas and 58 years among those prescribed DPP-4i. 40.1% of participants prescribed sulphonylureas were females while 41.3% prescribed DPP-4i were females. Baseline HbA1c [9.0% (75.4 mmol/mol) vs 8.7% (71.5 mmol/mol)] and history of MACE (16.5% vs 15.7%) were slightly higher, while baseline weight was lower (91.8 kg vs 98.6 kg) among those prescribed sulphonylureas. Prevalence of comorbidities across both groups was similar, as was medication prescribed at baseline; with the vast majority of those in both sulphonylurea and DPP-4i groups prescribed statins (73.7% vs 76.7%) and anti-hypertensives (64.1% vs 66.1%), respectively (Table 1).

**Table 1:** Baseline characteristics of included patients.

Characteristic Mean (SD) or n [%]	Sulphonylureas	DPP-4i	Overall
N	17,570	6267	23,837
Female	7047 [40.1]	2586 [41.3]	9633 [40.4]
Age Index (years)	60 (12)	58 (12)	59 (12)
**Townsend**			
1 (least deprived)	3513 [20]	1409 [22.5]	4922 [20.6]
2	3599 [20.5]	1231 [19.6]	4830 [20.3]
3	3889 [22.1]	1389 [22.2]	5278 [22.1]
4	3739 [21.3]	1251 [20]	4990 [20.9]
5 (most deprived)	2830 [16.1]	987 [15.7]	3817 [16]
Smoking (current)	2213 [12.6]	761 [12.1]	2974 [12.5]
MACE history (yes)	2891 [16.5]	986 [15.7]	3877 [16.3]
HbA1c (mmol/mol)	75.4 (19.9)	71.5 (15.9)	74.4 (19)
HbA1c (%)	9 (1.8)	8.7 (1.5)	8.7 (1.7)
Blood Pressure			
Diastolic (mmHg)	78.9 (9.4)	79.2 (9.2)	79 (9.3)
Systolic (mmHg)	134.5 (14.9)	134 (14.5)	134.4 (14.8)
Weight (kg)	91.8 (20.5)	98.6 (22.3)	93.6 (21.2)
BMI (Body Mass Index) (Kg/m^2^)*	31.9 (6.2)	34 (6.7)	32.5 (6.4)
**Comorbidities**			
Anaemias	2663 [15.2]	848 [13.5]	3511 [14.7]
Arrythmias	1648 [9.4]	530 [8.5]	2178 [9.1]
Cancer	1358 [7.7]	458 [7.3]	1816 [7.6]
CKD	1115 [6.3]	306 [4.9]	1421 [6]
Dementia	465 [2.6]	112 [1.8]	577 [2.4]
Epilepsy	407 [2.3]	128 [2]	535 [2.2]
Heart Failure	486 [2.8]	137 [2.2]	623 [2.6]
Hyperthyroid	167 [1]	50 [.8]	217 [.9]
Hypoglycaemias	416 [2.4]	115 [1.8]	531 [2.2]
Hypothyroid	826 [4.7]	300 [4.8]	1126 [4.7]
Liver	44 [.3]	26 [.4]	70 [.3]
Neuropathy	53 [.3]	18 [.3]	71 [.3]
Pancreatitis	295 [1.7]	71 [1.1]	366 [1.5]
Resp Disease	3918 [22.3]	1422 [22.7]	5340 [22.4]
Retinopathy	5211 [29.7]	1685 [26.9]	6896 [28.9]
SMI	382 [2.2]	131 [2.1]	513 [2.2]
**Concomitant medication**			
Statins	12,950 [73.7]	4805 [76.7]	17,755 [74.5]
Anti-hypertensives	11,256 [64.1]	4145 [66.1]	15,401 [64.6]
Antianginals	441 [2.5]	133 [2.1]	574 [2.4]
Anti-heart failure	1299 [7.4]	445 [7.1]	1744 [7.3]
Other lipid lowering drug	741 [4.2]	278 [4.4]	1744 [7.3]
Antiplatelets	5192 [29.6]	1465 [23.4]	6657 [27.9]
Anticoagulants	577 [3.3]	210 [3.4]	787 [3.3]
Antidepressants	3154 [18]	1261 [20.1]	4415 [18.5]
Antipsychotics	393 [2.2]	156 [2.5]	549 [2.3]
Antiobesity	213 [1.2]	134 [2.1]	347 [1.5]
Steroids –oral	931 [5.3]	252 [4]	1183 [5]
Thyroxine	1401 [8]	479 [7.6]	1880 [7.9]

DPP-4i = Dipeptidyl-peptidase-4 Inhibitors; MACE = Major adverse cardiovascular events; CKD = chronic kidney disease; SMI = several mental disease.

*BMI could not be calculated for 1.5% of the cohort due to absent height records.

**Resp Disease = Asthma and Chronic Obstructive Pulmonary Disease.

### Cardiovascular outcomes

#### Crude analysis

**Figure 1:**
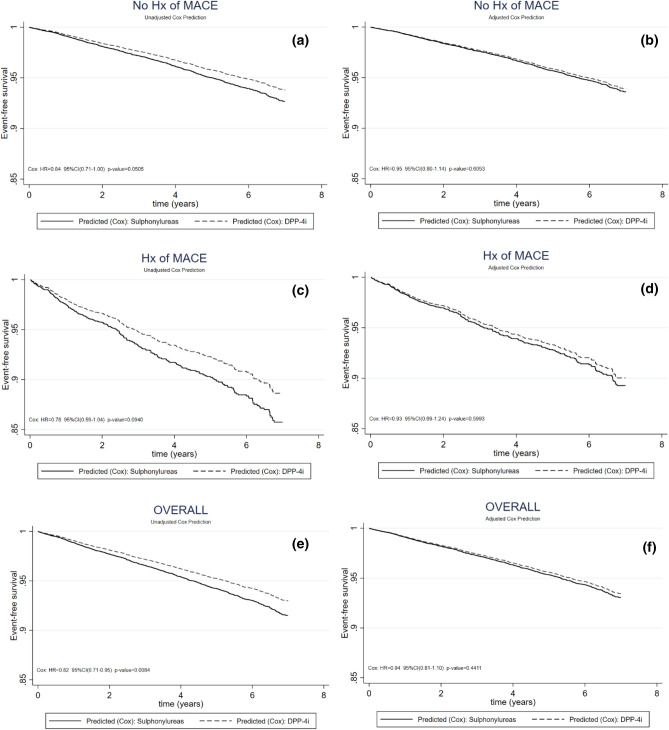
Cox regression survival predictions on major adverse cardiovascular events (MACE), comparing Sulphonylureas and DPP-4i cohorts for people with no history of MACE (No Hx of MACE), with history of MACE (Hx of MACE) and overall, unadjusted (left column: Fig. 1a, Fig. 1c, Fig. 1e) and adjusted for baseline covariates (right column: Fig. 1b, Fig. 1d, Fig. 1f.). Event-free survival over time, hazard ratios with 95% confidence intervals and p-values are reported.

**Figure 2:**
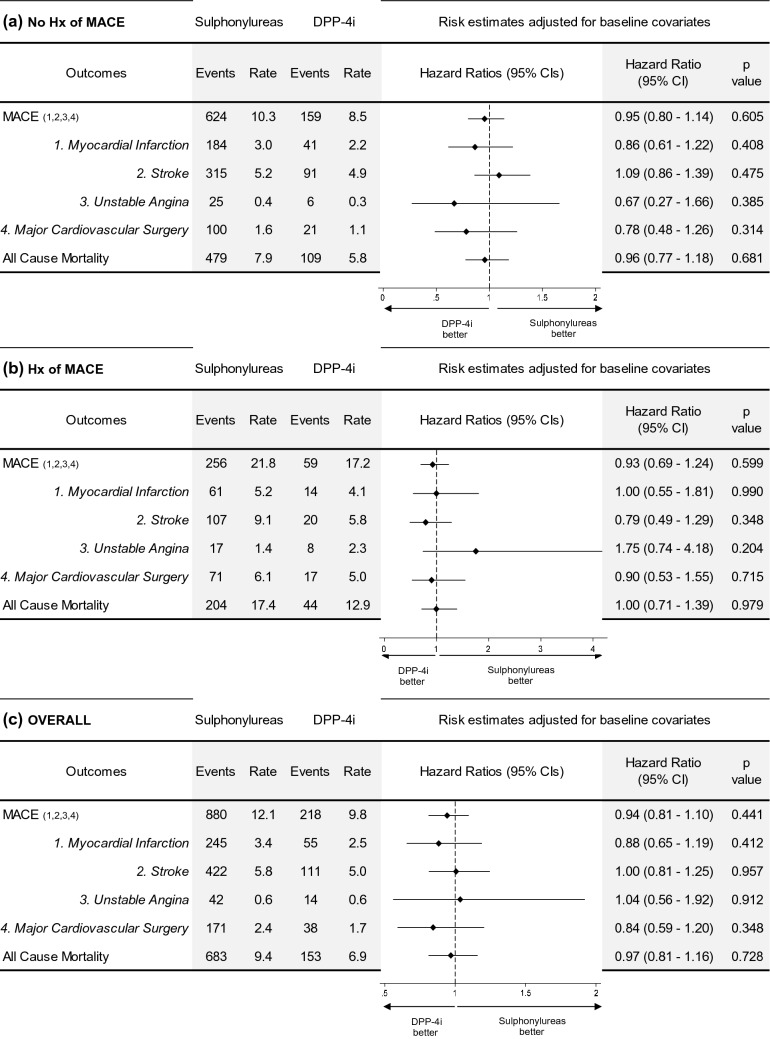
Adjusted hazard ratios on MACE, components of MACE and all-cause mortality, comparing Sulphonylureas and DPP-4i cohorts. Results stratified for people with no history of MACE (Fig. 2a), with prior history of MACE (Fig. 2b) and overall (Fig. 2c). Event rates are reported as number of events/1000 person-years.

Unadjusted survival predictions for MACE for the sulphonylureas and DPP-4i cohorts are shown in Fig. 1a, c and e (see Appendix B for the Kaplan Meier curves). The overall crude incidence (primary and secondary incidence combined) for MACE for those prescribed sulphonylureas was 12.1/1000 person-years at risk (PYAR) while it was 9.8/1000 PYAR for those prescribed a DPP-4i, as shown in Fig. 2a. Among those without a history of MACE, the primary incidence for MACE for those prescribed sulphonylureas was 10.3/1000 PYAR while it was 8.5/1000 PYAR for those prescribed a DPP-4i, as shown in Fig. 2b. For those with a pre-existing history of MACE, the secondary incidence for those prescribed sulphonylureas was 21.8/1000 PYAR while it was 17.2/1000 PYAR for those prescribed a DPP-4i, as shown in in Fig. 2c.

#### Adjusted analysis

The adjusted survival predictions on MACE for the sulphonylureas and DPP-4i cohorts are shown in Fig. 1b, d and f. After adjustment for potential confounding covariates such as baseline HbA1c, systolic blood pressure, weight, history of smoking, SMI and being prescribed concomitant cardiovascular medication such as statins and antihypertensives, the adjHR (adjusted hazard ratio) among individuals prescribed DPP-4i was 0.94 (95% C.I. 0.81–1.10) for the overall cohorts, suggesting no significant difference (Fig. 2c). In the cohort without a history of MACE, the adjHR for primary MACE among individuals prescribed DPP-4i vs sulphonylureas was 0.95 (95% C.I. 0.80–1.14), indicating no statistically significant difference. Similarly, for those with a pre-existing history of MACE, the adjHR for secondary incidence of MACE among individuals prescribed DPP-4i was 0.93 (95% C.I. 0.69–1.24), also indicating no statistically significant difference. The sensitivity analysis undertaken, combining all-cause mortality as part of the MACE definition, did not result in any change in interpretation for the adjusted estimates (Appendix C).

The incidence for the individual components comprising our MACE definition for those both with and without history of pre-existing MACE, are displayed in Fig. 2. This indicated there was no significant difference in frequency of MI, stroke, major cardiovascular surgery or diagnosis of unstable angina between those prescribed DPP-4i and sulphonylureas. Equally there was no significant difference for our secondary outcome, rates of all-cause mortality between groups.

### Additional and subgroup analysis

**Figure 3:**
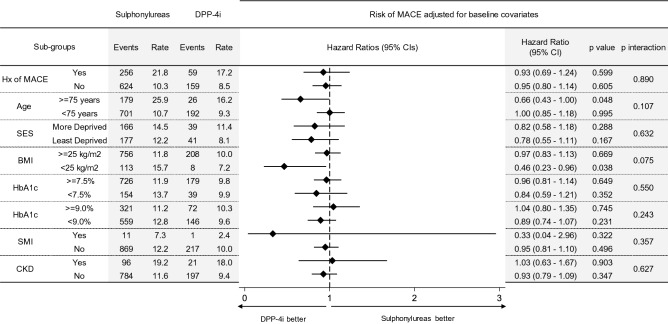
Subgroup analysis. Adjusted hazard ratios on MACE, comparing Sulphonylureas and DPP-4i cohorts by groups considering history of MACE, age, socio-economic status (SES), body mass index (BMI), HbA1c, several mental illness (SMI) and chronic kidney disease (CKD). Event rates are reported as number of events/1000 person-year.

We undertook several pieces of additional subgroup analysis to examine cardiovascular outcomes across several important risk strata (Fig. 3). No evidence was found of any deviation in the incidence of MACE among these groups, except among those aged ≥ 75 years and with a BMI < 25 kg/m^2^ where there a suggestion of lower MACE incidence among those prescribed DPP-4 inhibitors.

## Discussion

### Summary of findings

In this retrospective cohort study undertaken in patients with T2DM in routine clinical practice, we found that patients initiating a DPP-4i as add-on to metformin demonstrated a similar risk of major cardiovascular events (MACE) compared to those prescribed sulphonylureas. We showed that this finding was consistent among both those with and without a previous history of MACE, as well as among several pertinent population subgroups such as those from contrasting socio-economic backgrounds, differing baseline HbA1c and weight, as well as history of CKD and SMI at initiation. There was a suggestion that DPP-4i was more protective in older patients aged ≥ 75 years against MACE and with a BMI < 25 kg/m^2^, but as this is a subgroup analysis only, further research is warranted here.

### Comparison with literature

Four phase 3 randomised trials initially compared DPP-44i to placebo and established its cardiovascular safety, though it remained unclear whether it had a better cardiovascular safety profile than other anti-diabetics^10,22–24^. Additionally, there is always a challenge in translating trial evidence into clinical practice and those recruited to trials are not always reflective of patients in the “real world” utilising these treatments. For example, in the TECOS cardiovascular trial for the DPP-4 inhibitor, sitagliptin, the patients recruited had better controlled diabetes at baseline (compared to “real world” populations)^23^. They had a HbA1c between 48 mmol/mol (6.5%) and 64 mmol/mol (8.0%) and were excluded if they had a history of two or more episodes of hyperglycaemia in the previous 12 months^23^. In our study, the mean Hba1c at baseline was 74.4 mmol/mol in the cohort, and 2.2% recorded a history of hypoglycaemia prior to initiation during which period they had only been on metformin for glycaemic control. Such a profile is more typical of patients that receive these add-on treatments in clinical practice.

In addition to placebo-controlled trials, the CAROLINA trial was also undertaken which was the first head-to-head trial of a DPP-4i, linagliptin against sulphonylurea, glimepiride. It was undertaken among a cohort of less advanced, type 2 diabetes patients^10^. In CAROLINA, outcomes were compared among those prescribed linagliptin vs glimepiride, that were already on metformin or another first-line treatment; a more accurate reflection of clinical practice. They found that risk of MACE (defined as 3P-MACE including cardiovascular death, nonfatal myocardial infarction, or nonfatal stroke) was similar with both treatments; HR = 0.98 [95% C.I., 0.84–1.14]. They also found no increased risk of hospitalisation for heart failure with DPP-4i, a cited concern previously^10,25^. Despite our use of an altered definition for MACE that our real-world dataset could support, our findings were similar in this population free of MACE at baseline; HR, 0.92 [95% C.I., 0.77–1.10]. CAROLINA excluded patients on other DPP-4is or sulphonylureas, on insulin and with more established cardiovascular disease^10^. In our study, we examined the DPP-4 class as whole against sulphonylureas in real-world patients both with and without a history of MACE. We demonstrated similar cardiovascular event rates with both treatments, among those with a history of MACE at baseline as well as without. We also found similar outcomes with both treatments for all-cause mortality, and across several vulnerable subgroups by HbA1c, socioeconomic status and a history of SMI and chronic kidney disease.

Previous database studies undertaken using data from clinical practice and registries where DPP-4i and sulphonylureas have been compared have also shown similar risks of cardiovascular outcomes with both. However a major limitation of these studies has been limited follow-up, samples size, inadequate comparators and most commonly, an inability to adjust for key confounders such as HbA1c, weight and smoking status at baseline^12,13,26^.

There have been several updates to treatment algorithms in recent years for T2DM, following emergence of newer DPP-4i, SGLT-2 and GLP-1 analogues. Agents in the latter two classes have also demonstrated evidence of cardiovascular benefits in Phase 3 clinical trials^11^, however choosing add-on therapy to metformin has become increasingly complex for prescribers. Indeed, the American Diabetes Association (ADA) and the European Association for the Study of Diabetes (EASD) guidelines break down recommendations for add-on according to costs, risk of adverse events, as well as history of complications^27^. For example, it specifically recommends that among those individuals where there is need for intensification when metformin alone is inadequate and there is either a “compelling need to minimize hypoglycaemia, or a “ need to minimize weight gain”, then add-on therapy should consider DPP-I among other options but importantly avoid sulphonylureas. Equally, the guidelines also note specifically that when “cost is a major issue” that sulphonylureas are considered as an option. They also advocate, preferential use of GLP-1 analogue or SGLT-2 inhibitors with proven cardiovascular benefit as add-on to metformin, among those with established or at high-risk of cardiovascular disease. Contrastingly, UK NICE guidance does not discriminate between add-on treatments except for GLP-1 analogues which are only recommended in those with a BMI ≥ 35 kg/m^2^ or as an alternative to those needing insulin^28^. This may explain why trends in UK clinical practice still suggest, that in those individuals both with and without baseline cardiovascular disease, DPP-4i and sulphonylureas remain the most commonly prescribed add-on treatments to metformin for the last decade^2^. As a consequence, we were unable to extend our analysis as intended, to examine risk of MACE among those prescribed SGLT-2i and GLP-1 analogues as add-on to metformin as well. Due to small sample sizes, there was insufficient power to make meaningful conclusions from UK primary care data with regards to their longer-term effectiveness in this context^5^.

Several studies have compared DPP-4i vs sulphonylureas for other outcomes and these have consistently demonstrated similar glycaemic reductions, weight neutral/loss with DPP-4i and gain with sulphonylureas and a consistently higher risk of hypoglycaemias with sulphonylureas^3^. Indeed in CAROLINA, 1 or more episodes of hypoglycemia occurred in 320 (10.6%) participants in the linagliptin group but in 1132 (37.7%) in the glimepiride group, highlighting a significant disparity^10^. Previous concerns with DPP-4i regarding pancreatitis have also been largely allayed in recent studies, and more recently, evidence supporting their glycaemic efficacy in older adults has emerged as well^4^. This study has now shown, using real-world data, that cardiovascular outcomes in clinical practice appear similar with both treatments. Despite this growing evidence supporting benefits of DPP-4i mainly from a safety and tolerability point of view, they do however remain more costly than sulphonylureas, meaning setting-specific economic evaluations remain essential in guiding selection.

### Strengths and limitations

There are several strengths to this study. The study used real-world data from UK primary care where prescribing for T2DM and add-on therapy are managed, ensuring data are comprehensive and reflect true clinical practice. We examined cardiovascular outcomes, among a cohort of patients in routine clinical practice who were already on metformin and at similar stages in their disease management trajectories, thus reducing the potential for confounding by indication^29^. This makes populations prescribed sulphonylurea and DPP-4i more similar at baseline, as was found in our analysis of baseline characteristics. There have been few head-to-head clinical trials that have compared the effects of different anti-diabetics on cardiovascular event rates in T2DM, among both patients with and without established cardiovascular disease, hence this study addresses an importance evidence gap. Despite growing evidence to support cardiovascular benefits of some SGLT-2 inhibitors and GLP-1 analogues in those with higher baseline cardiovascular risk^11,30^, DPP-4i and sulphonylureas remains the most widely prescribed treatments in the UK due to both cost and practical reasons, given need for subcutaneous administration for GLP-1 analogues^1^. Hence, evidence comparing DPP-4 inhibitors and sulphonylureas remains clinically highly relevant. Our samples size allowed for robust outcome and exploratory subgroup analyses.

There are notable limitations. We were unable to analyse cardiovascular outcomes for other add-on treatments to metformin such as SGLT-2 inhibitors and GLP-1 analogues due to lack of sample size. Our subgroup analysis, which raised the possibility of a protective cardiovascular effect with DPP-4i in older adults and those with BMI < 25 kg/m^2^, needs further investigation as the sample size is small and given higher prevalence of comorbidity in this group^31^. As with any observational study, despite extensive adjustment for potential confounders including key ones excluded in several previous studies, a risk of residual and unmeasured confounding remains.

## Conclusions

Among patients with T2DM in clinical practice, both with and without a history of MACE, DPP-4i exhibited a similar risk of incident MACE including myocardial infarction, stroke, major cardiovascular surgery, and diagnosis of unstable angina as well as all-cause mortality, when compared to sulphonylureas. This means that cardiovascular considerations alone cannot be the basis for choosing between either therapy. Other existing efficacy data also show equivalence of DPP-4is and sulphonylureas. This means the decision between both should largely be based on local economic considerations, given that DPP-4i remain more costly however demonstrate no propensity for weight gain and are superior in terms of clinical safety, with respect to hypoglycaemias.

### Patient and public involvement

The Lay ADvice on Diabetes and Endocrine Research (LADDER) Panel based in Sheffield provided PPI input into this project. This consists of a panel made up of patients, carers and people with an interest in diabetes or an endocrine condition. They discussed the entire project proposal provided them to them in lay language and provided in depth feedback on the project – in terms of core aims, objectives and methodology employed while also identifying areas for research not previously considered. They have also kindly agreed to aid with dissemination of findings when project is complete.

## Supplementary Information

Supplementary Information.

## Data Availability

Data were analysed under THIN license. THIN data are not publicly available.

## Abbreviations

MACE: Major adverse cardiovascular events
adjHR: Adjusted hazard ratio
DPP-4i: Dipeptidyl-peptidase-4 Inhibitors
T2DM: Type-2 diabetes mellitus
IMRD: IQVIA Medical Research Data
SMI: Severe mental illness
HR: Hazard ratio
CKD: Chronic kidney disease
BMI: Body mass index
PYAR: Person-years at risk
ADA: American Diabetes Association
EASD: European Association for the Study of Diabetes
