# Supplementary material for: Cardiovascular outcomes of type 2 diabetic patients treated with DPP‑4 inhibitors versus sulphonylureas as add-on to metformin in clinical practice
Source: Sci Rep. 2021 Dec 13;11:23826. doi: 10.1038/s41598-021-02670-9 (PMC8668905; doi:10.1038/s41598-021-02670-9)
Supplement: Supplementary file 1 — Supplementary Information. [file 41598_2021_2670_MOESM1_ESM.docx]

# Supplemental Material

## Appendix A: Flowchart of the study sample

Individuals between 2008-2017 who were on metformin and went onto have second-line add on treatment, fulfilling inclusion and exclusion criteria of the study **(N=32,820)**

**Removed** cases with other second-line add on treatment than Sulphonylureas or DPP-4 inhibitors:

Thiazolidinediones (N=2,363, **7.2%** of 32,820)

Insulin (N=984, **3.0%** of 32,820)

GLP-1 analogues (N=605, **1.8%** of 32,820)

SGLT-2 inhibitors (N=475, **1.4%** of 32,820)

Cases **removed** due to missing data*, for performing a complete case analysis:

Missing data in HbA1c (N= 2,665, **8.1%** of 32,820)

Missing data in systolic blood pressure (N= 560, **1.7%** of 32,820)

Missing data in weight (N= 1,331, **4.1%** of 32,820)

Study Cohort (**N=** **23,837)**

*(*) Smoking is a binary variable showing current and non-current smokers (i.e., ex-smoker or never smoker) and missing data on smoking were recoded as non-current smoker. This approach resulted in a prevalence of current smoker consistent with others reported elsewhere ^1,2^.*

References:

*Patorno E, Goldfine AB, Schneeweiss S, Everett BM, Glynn RJ, Liu J, Kim SC. Cardiovascular outcomes associated with canagliflozin versus other non-gliflozin antidiabetic drugs: population based cohort study. Bmj. 2018 Feb 6;360.*

*2 Hippisley-Cox J, Coupland C. Diabetes treatments and risk of heart failure, cardiovascular disease, and all cause mortality: cohort study in primary care. bmj. 2016 Jul 13;354.*

## Appendix B: Kaplan Meier curves with risk tables for Figure 1.


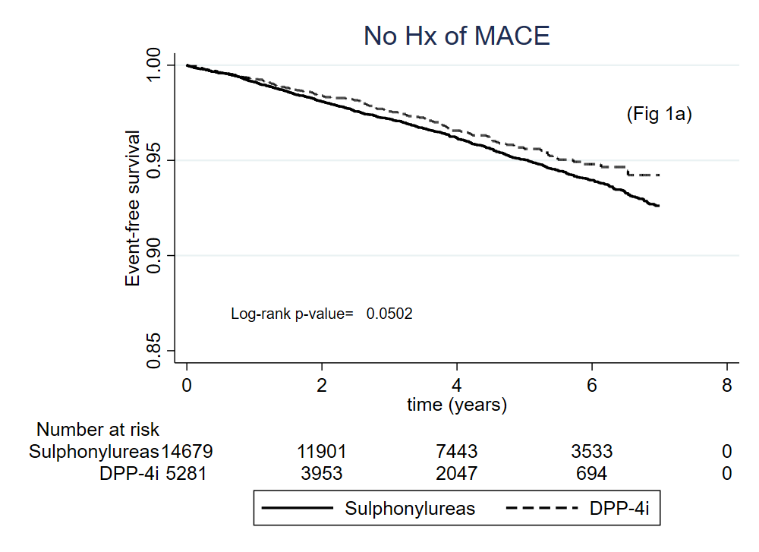

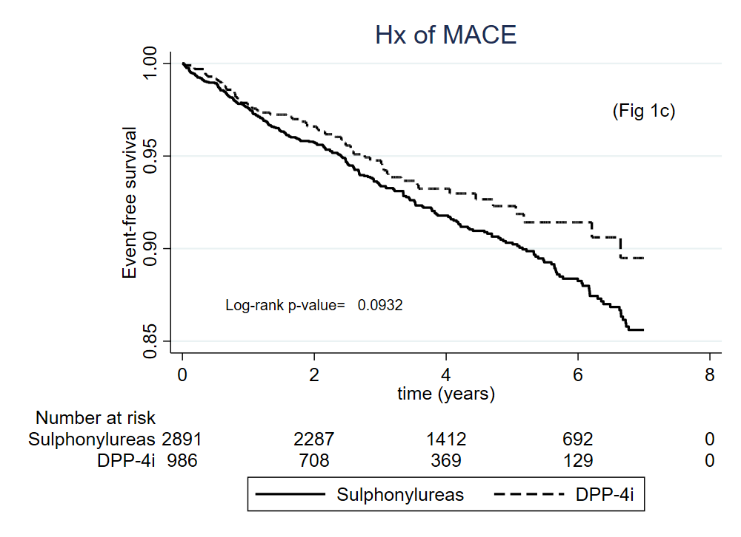

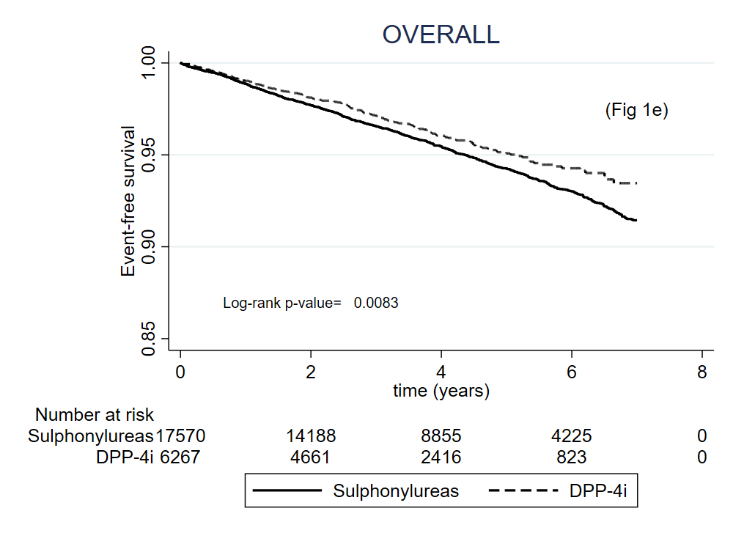


*This figure was drawn with Stata/MP version 16.*

## Appendix C: Sensitivity Analysis for Figure 2 (including all-cause mortality in MACE)


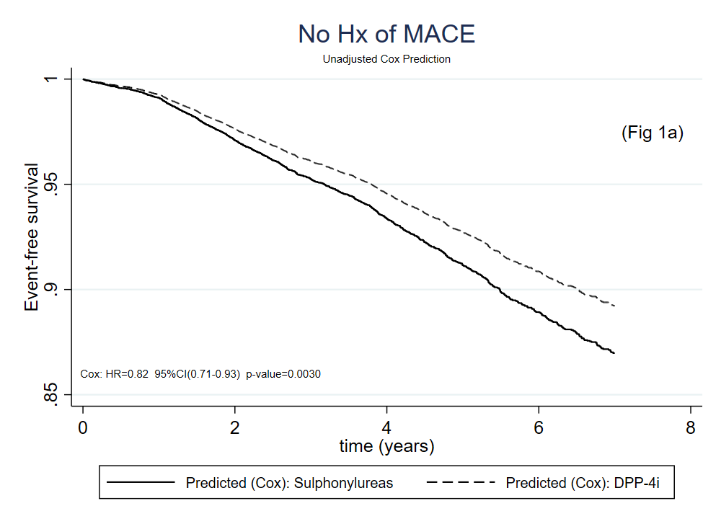

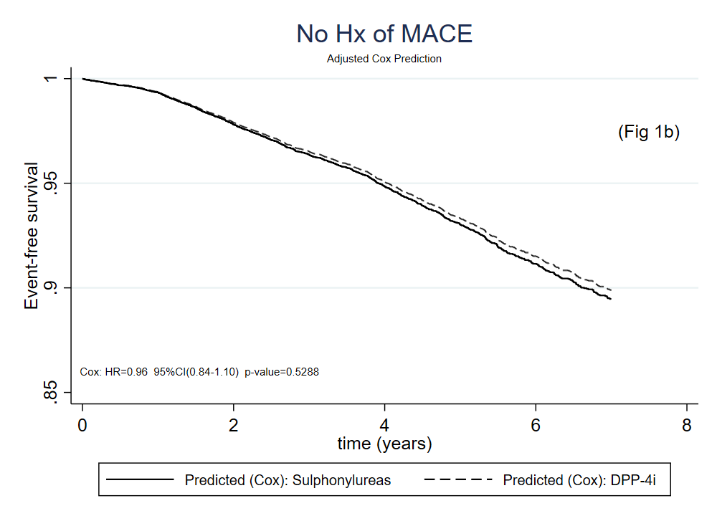

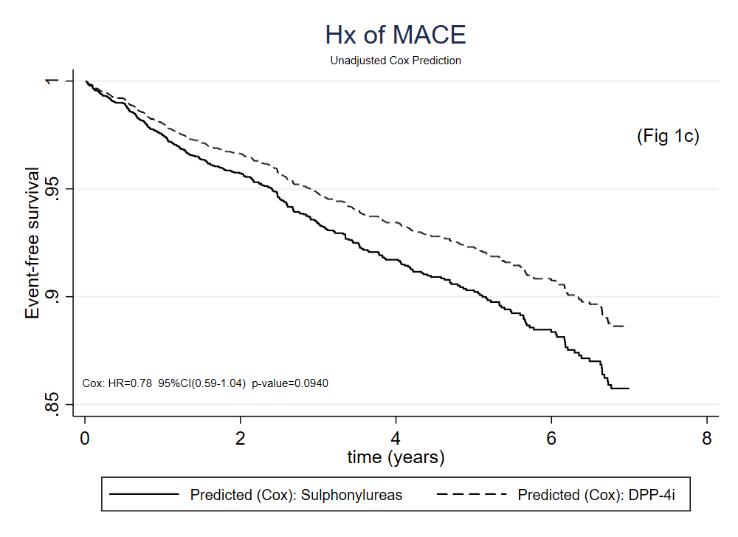

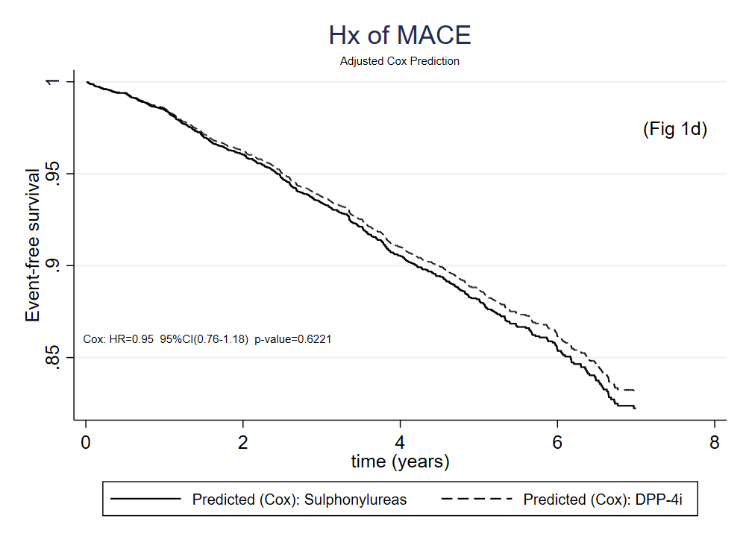

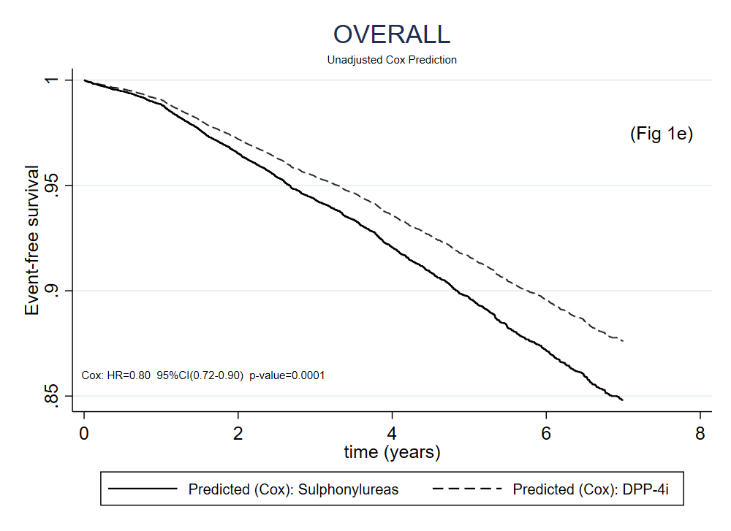

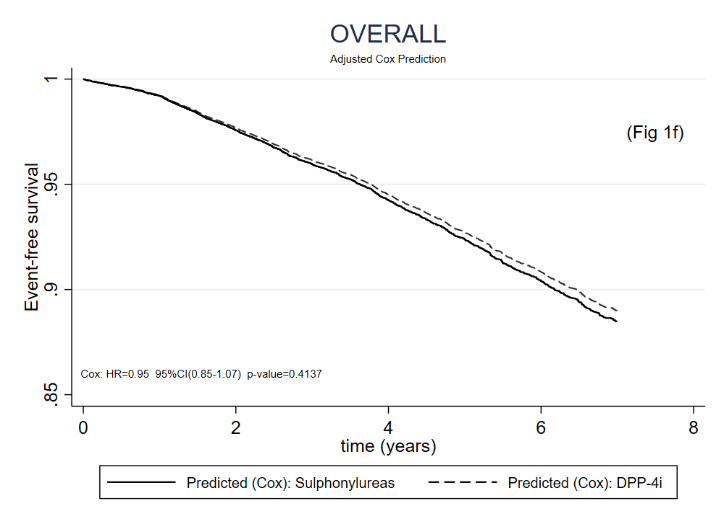


*This figure was drawn with Stata/MP version 16.*

Sensitivity Analysis for Figure 1. Cox regression survival predictions on major adverse cardiovascular events (MACE) and all-cause mortality (composite outcome), comparing Sulphonylureas and DPP-4i cohorts for people with no history of MACE (No Hx of MACE), with history of MACE (Hx of MACE) and overall, unadjusted (left column: Fig 1a, Fig 1c, Fig 1e) and adjusted for baseline covariates (right column: Fig 1b, Fig 1d, Fig 1f). Event-free survival over time, hazard ratios with 95% confidence intervals and p-values are reported.
